# 

**Published:** 1890-09

**Authors:** 


The editor of the JOURNAL has no connection whatever with "The Types of Successful Men," and all communication in regard to that work should be addressed to Dr. J. J. Tobin.

---

The Secretary of the Texas State Medical Association is authorized to state that the Transactions of the Association will be completed and ready for delivery early in next month.

